# Mitochondrial Inhibitory Factor 1 (IF1) Is Present in Human Serum and Is Positively Correlated with HDL-Cholesterol

**DOI:** 10.1371/journal.pone.0023949

**Published:** 2011-09-14

**Authors:** Annelise Genoux, Véronique Pons, Claudia Radojkovic, Florence Roux-Dalvai, Guillaume Combes, Corinne Rolland, Nicole Malet, Bernard Monsarrat, Frédéric Lopez, Jean-Bernard Ruidavets, Bertrand Perret, Laurent O. Martinez

**Affiliations:** 1 INSERM, UMR1048, Institut de Maladies Métaboliques et Cardiovasculaires, Toulouse, France; 2 Université de Toulouse, UPS, Toulouse, France; 3 CHU Toulouse, Hôpital Purpan, Toulouse, France; 4 CNRS, IPBS (Institut de Pharmacologie et de Biologie Structurale), Toulouse, France; 5 INSERM, UMR1027, Toulouse, France; University of Tor Vergata, Italy

## Abstract

**Background:**

Mitochondrial ATP synthase is expressed as a plasma membrane receptor for apolipoprotein A-I (apoA-I), the major protein component in High Density Lipoproteins (HDL). On hepatocytes, apoA-I binds to cell surface ATP synthase (namely ecto-F_1_-ATPase) and stimulates its ATPase activity, generating extracellular ADP. This production of extracellular ADP activates a P2Y_13_-mediated HDL endocytosis pathway. Conversely, exogenous IF1, classically known as a natural mitochondrial specific inhibitor of F_1_-ATPase activity, inhibits ecto-F_1_-ATPase activity and decreases HDL endocytosis by both human hepatocytes and perfused rat liver.

**Methodology/Principal Findings:**

Since recent reports also described the presence of IF1 at the plasma membrane of different cell types, we investigated whether IF1 is present in the systemic circulation in humans. We first unambiguously detected IF1 in human serum by immunoprecipitation and mass spectrometry. We then set up a competitive ELISA assay in order to quantify its level in human serum. Analyses of IF1 levels in 100 normolipemic male subjects evidenced a normal distribution, with a median value of 0.49 µg/mL and a 95% confidence interval of 0.22–0.82 µg/mL. Correlations between IF1 levels and serum lipid levels demonstrated that serum IF1 levels are positively correlated with HDL-cholesterol and negatively with triglycerides (TG).

**Conclusions/Significance:**

Altogether, these data support the view that, in humans, circulating IF1 might affect HDL levels by inhibiting hepatic HDL uptake and also impact TG metabolism.

## Introduction

The role of High Density Lipoproteins (HDL) in protecting against atherosclerosis is now well established but the mechanism supporting this is still debated. The original proposal, supported by a wide range of evidences, attributes this atheroprotective effect of HDL particles to their metabolic role in “reverse cholesterol transport’ (RCT), a process which carries cholesterol from peripheral tissues back to the liver for further catabolism. In addition, HDL have a direct atheroprotective effect on the vascular wall through anti-inflammatory, antioxidant and antithrombotic actions involving *inter alia* the production of nitric oxide (NO). Hepatic uptake of HDL particles is a key step in RCT [1]. Two distinct mechanisms of HDL-cholesterol uptake have been described in hepatocytes: i) selective cholesterol uptake, a mechanism through which cholesterol (free or esterified) is taken up while the protein components of the HDL particle are not; ii) HDL endocytosis resulting in the uptake and degradation of HDL holoparticle [2]. In contrast to the well-established role of the Scavenger receptor class B member I (SR-BI) in selective cholesterol uptake by hepatocytes [3], a distinct pathway seems to be involved in HDL holoparticle endocytosis [4]. A likely candidate mediating HDL holoparticle endocytosis is the cell surface complex related to mitochondrial ATP synthase (namely ecto-F_1_-ATPase) [5]. Apolipoprotein A-I (apoA-I) binding to ecto-F_1_-ATPase stimulates extracellular ATP hydrolysis to ADP and the latter specially activates the nucleotide receptor P2Y_13_ resulting in HDL holoparticle endocytosis [6], [7]. We and others have described that P2Y_13_-knock out mice displayed altered HDL hepatic catabolism and impaired RCT [8], [9], strongly suggesting that this ecto-F_1_-ATPase/P2Y_13_-mediated HDL endocytosis pathway might play an important role in HDL metabolism *in vivo*.

Thus, regulation of ecto-F_1_-ATPase activity, which generates extracellular ADP, might directly impact on P2Y_13_ activation and subsequent hepatic HDL uptake. In mitochondria, the endogenous inhibitory protein IF1 regulates the ATPase activity of F_O_F_1_-ATP synthase [10]. IF1 is a basic amphiphilic mitochondrial protein of 81 amino acids (NCBI Reference Sequence: NP_057395.1 NM_016311.4) with a significant degree of homology in various species [11]. Data from high-resolution crystallography have shown that IF1 binds through its N-terminal region at the interface between α_DP_- and β_DP_-subunits of the F_1_ catalytic sector of mitochondrial ATP synthase, thereby blocking rotary catalysis [12].

Therefore, IF1 regulates mitochondrial energetic functions under both physiological and pathological conditions, such as ischemic injury, by maintaining mitochondrial ATP levels when ATP synthase switches from ATP synthesis to ATP hydrolysis [13], [14]. We and others have also reported that exogenous IF1 could specifically bind to ecto-F_1_-ATPase on hepatocytes and endothelial cells and reduce its hydrolytic activity [6], [15], [16]. Interestingly, exogenous IF1 was able to reduce by about 50% HDL uptake by perfused rat liver [6], suggesting that the inhibitory effect of IF1 on ecto-F_1_-ATPase activity might impact on HDL metabolism. More recently, endogenous IF1 has been identified on the surface of both endothelial cells and hepatocytes [16], [17], [18], [19], suggesting that endogenous IF1 might also be a potent regulator of HDL metabolism.

In this study, we describe for the first time the presence of endogenous IF1 in human serum and further analyze the correlation between IF1 and serum lipid levels in 100 normolipemic subjects.

## Results

### Identification of IF1 in human serum

**Figure 1:**
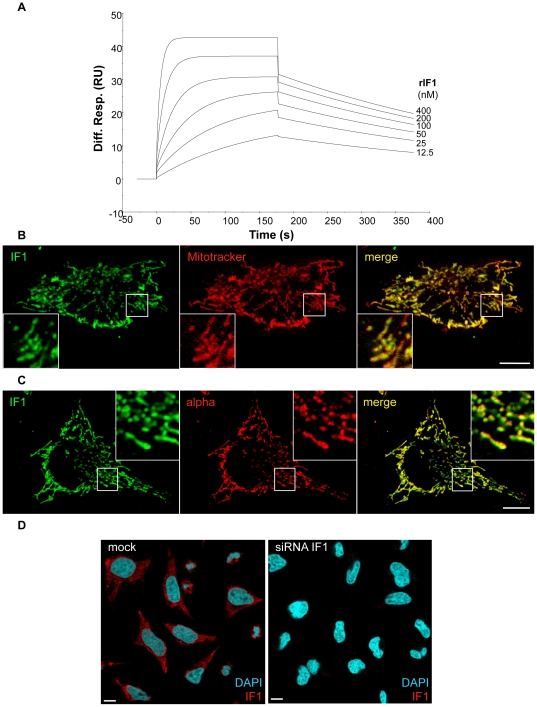
Characterization of polyclonal anti-IF1 antibody. (A) Sensorgrams are representative of specific interactions (differential response) while nonspecific binding (with no protein immobilized) was deduced. rIF1 was injected at a concentration ranging from 12.5 to 400 nM. Results are expressed as resonance units (RU) as a function of time in seconds. The apparent kinetic constants of the interaction were k_a_ = 3.87e^5^ M^−1^ s^−1^, k_d_ = 2.37e^−3^ s^−1^, and K_D_ = 6.12e^−9^ M. (B) HeLa cells were incubated or not with Mitotracker for 30 min at 37°C. Cells were then fixed and processed for immunofluorescence using anti-IF1 antibody. (C) HeLa cells were fixed and processed for immunofluorescence using both anti-IF1 and anti-alpha subunit of ATP synthase (alpha) antibodies. In (B) and (C), the merged images demonstrate perfect overlap between IF1 and both Mitotraker and alpha chain of mitochondrial ATP synthase. (D) HeLa cells were treated with siRNAs against IF1, fixed and then processed for immunofluorescence using anti-IF1 antibody and DAPI for nuclear stain. (B–D) Scale bar indicates 10 µm.

A polyclonal antibody was raised against human recombinant IF1 (rIF1) and purified as described in the Materials and Methods section. Interaction of this anti-IF1 antibody with rIF1 was controlled by Surface Plasmon Resonance (SPR) analysis (Fig. 1A). In these conditions, rIF1 specifically bound to anti-IF1 immobilized onto SPR chips (K_D_ = 6.1 nM). To further evaluate the specificity of anti-IF1, we performed immunofluorescence experiments in HeLa cells. Immunolabeling with anti-IF1 antibody showed a characteristic mitochondrial pattern (Fig. 1B). This was further confirmed by colocalization of IF1 staining with mitochondrial markers such as MitoTracker (Fig. 1B) or the alpha subunit of ATP synthase (Fig. 1C). This staining is completely lost when cells were treated with siRNA against IF1 (Fig. 1D), highlighting the specificity of anti-IF1 antibody. Altogether, these data demonstrate the specificity of the anti-IF1 antibody toward recombinant and endogenous IF1 protein.

**Figure 2:**
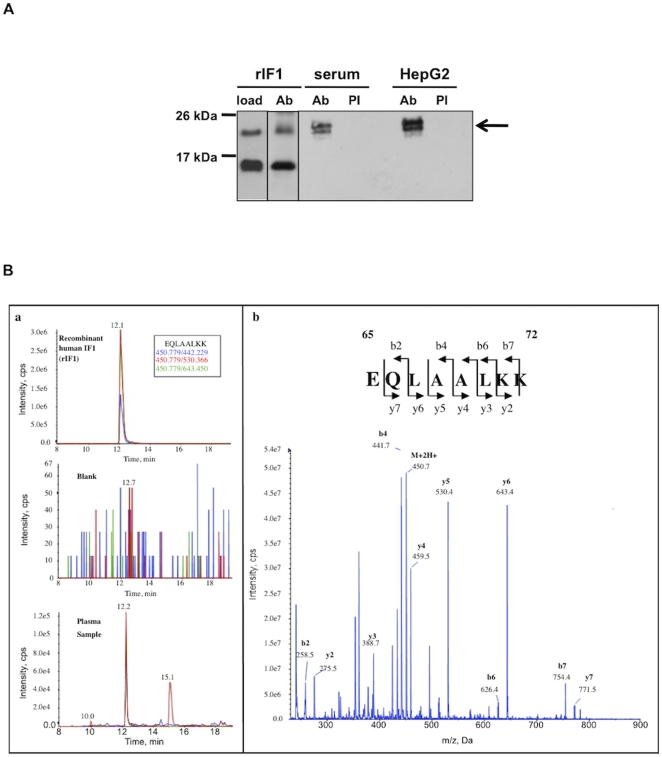
Identification of IF1 in human serum. (A) Recombinant IF1 (rIF1) or low-abundance protein-enriched serum or HepG_2_ cell lysates were subjected to immunoprecipitation with pre-immune antibodies (PI) or anti-IF1 antibodies (Ab). Analysis was performed by Western blotting using antibodies against IF1. On the left panel, 1 µg rIF1 was loaded as a control of molecular weight. Arrow points at 2 bands immunoprecipitated with specific anti-IF1 antibody. (B) (a) Multiple Reaction Monitoring of EQLAALKK peptide of rIF1 (high panel), blank run before the serum sample (middle panel) and serum sample (low panel). Note the scale difference between rIF1, the serum sample and blank run. Three parent/fragment transitions are monitored for each peptide (represented in three different colors). (b) Manually annotated MS/MS fragmentation spectra of EQLAALKK peptide of human IF1 from serum sample.

This antibody was then used to immunopurify IF1 from a pool of serum enriched in low-abundance proteins and from HepG_2_ cell lysate. Proteins from the immunopurified fractions were then separated on a polyacrylamide gel and immunoblotted with anti-IF1 antibody. As compared to immunoprecipitation performed using pre-immune IgG, a specific signal for IF1 was detected in anti-IF1 immunopurified fractions from both human serum and HepG_2_ cell lysate (Fig. 2A). Interestingly, in these conditions, IF1 from serum and hepatocytes appeared as a double band, at the size of a dimer whereas under the same conditions, purified rIF1 is immunoprecipitated both as a monomer and a dimer. However, upon longer exposure time, a faint specific band at the size of the monomer can also be detected in immunoprecipitated samples from both serum and HepG_2_ cell lysate (data not shown). This result suggested that IF1 was present in human serum and mostly found as a dimeric form, like in hepatocytes.

In order to confirm the presence of IF1 in human serum, a targeted proteomics method has been implemented: the detection of IF1 by Multiple Reaction Monitoring (MRM, also called Selected Reaction Monitoring, SRM) mass spectrometry has been optimized using the rIF1. Four peptides (EQLAALK, EQLAALKK, LQKEIER, HHEEEIVHHK) were monitored and the corresponding MS/MS sequences were acquired. The same method has then been used on human serum pool sample previously enriched for minor proteins using ProteoMiner™ protocol. The comparable MRM traces and MS/MS fragmentation spectra for all four peptides in rIF1 and serum sample demonstrate the presence of IF1 in human serum. This was confirmed by a protein database search with serum sample spectra using Mascot software. The results obtained for one of these peptides are shown in figure 2B. Altogether, these data unambiguously demonstrated the presence of IF1 in human serum.

### Quantification of IF1 serum levels in the general population and correlation with serum lipid parameters

**Figure 3:**
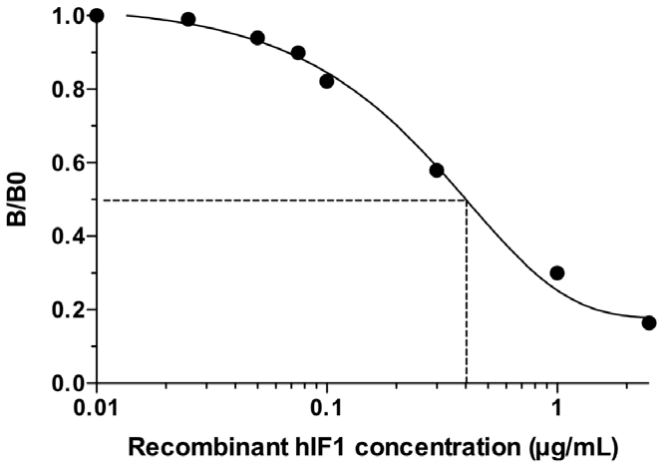
Typical standard curve used for IF1 quantification. Competitive ELISA was performed using rIF1-coated plates (0.5 µg/mL) and biotinylated anti-IF1 polyclonal antibody pre-incubated with different dilutions of rIF1 (0 to 2.5 µg/mL). The colorimetric signal (OD 450 nm–OD 570 nm) is expressed as a part of the maximum signal obtained without rIF1, B/B0. The standard curve shows that fifty percent displacement of binding was obtained at a concentration of 0.4 µg/mL rIF1.

On the basis of the use of anti-IF1 antibody, a competitive ELISA was developed as described in Materials and Methods section to quantify IF1 in human serum. Figure 3 displays a typical standard curve used for the ELISA, ranging from 0 to 2.5 µg IF1/mL. Fifty percent displacement was obtained at a concentration of 0.4 µg/mL. Repeatability (within the same day) and reproducibility (10 measurements over a 6-month period) gave variation coefficients of 6–7%. An average recovery of 96.8% was measured in dilution experiments. Resistance to freezing and thawing was assessed in different occasions with a 95%–103% agreement between measurements. With regard to possible correlations of IF1 with HDL markers, competition experiments with purified apolipoproteins A-I and A-II indicated no cross reactivity in this immunoassay (data not shown).

**Figure 4:**
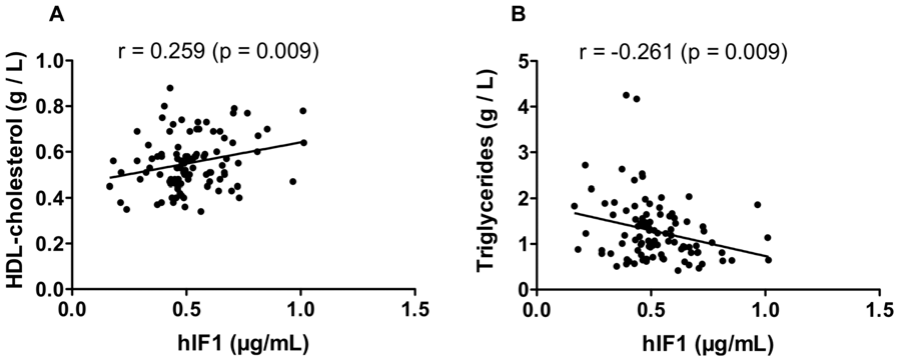
Correlation between IF1 levels and HDL-C (panel A) and TG (panel B). All individual data of measurements in 100 normolipemic male subjects are represented. Regression coefficients (r) and statistical significance (p) are given.

IF1 concentrations were measured among 100 healthy normolipemic male subjects, randomly selected from a population study. Among others, mean parameters (means ± SD) for this sub-population were: age, 59.1±8,8 y; body mass index (BMI), 26.8±3.5 kg/m^2^; systolic blood pressure, 136.9±16.0 mm Hg, glycaemia, 5.5±1.1 mmol/L; cholesterol, 2.3±0.37 g/L and triglycerides, 1.23±0.75 g/L. Regarding all investigated characteristics, this sub-population did not differ from the whole population study. IF1 concentrations displayed a normal distribution with an average value of 0.52±0.16 µg/mL, a median at 0.49 µg/mL and a 95% confidence interval of 0.22–0.82 µg/mL. Serum lipid levels were compared from both sides of IF1 median. In subjects with elevated IF1, HDL-cholesterol was significantly higher and triglycerides (TG) or VLDL-cholesterol were lower, while there was no difference regarding total or LDL-cholesterol (Table 1). Moreover, correlations between IF1 serum levels and HDL-cholesterol and TG are displayed with all individual data points in Figure 4A and 4B respectively. Correlation analysis demonstrated that IF1 serum level was positively associated with HDL-cholesterol (r = 0.259, p = 0.009) and negatively associated with TG (r = −0,261, p = 0.009). No difference was recorded according to IF1 distribution in other investigated parameters, such as glucose level, cigarette smoking, alcohol consumption or physical activity (data not shown). These data support the view that IF1 might have an impact on the metabolism of both HDL and TG-rich lipoproteins.

**Table 1:** Correlations between IF1 levels and lipid parameters in serum.

	Below IF1 median (n = 50)	Above IF1 median (n = 50)	p
Total Cholesterol (g/L)	2.30 (0.37)	2.31 (0.35)	0.87 (NS)
HDL-C (g/L)	0.53 (0.12)	0.58 (0.11)	0.04 *
LDL-C (g/L)	1.48 (0.34)	1.51 (0.29)	0.59 (NS)
VLDL-C (g/L)	0.29 (0.02)	0.22 (0.01)	0.01 *
Triglycerides (g/L)	1.45 (0.82)	1.11 (0.42)	0.03# *

Serum lipid levels were compared from both sides of IF1 median (IF1 median = 0.49 µg/mL). Values are expressed as means and (SD); n = 100 normolipemic male subjects from the study population.

*Indicates significant difference (p<0.05; # after log transformation).

## Discussion

For the first time, we unambiguously demonstrated the presence of IF1 in human serum using a recently developed targeted proteomics approach allowing the identification of low-abundance proteins with high sensitivity and specificity [23]. This is in line with recent studies reporting ectopic localisation of endogenous IF1 at the cell surface of different cell types including endothelial cells [17] and hepatocytes [16], [18], [19]. Thus, IF1 is not strictly confined within the mitochondria but is ectopically expressed at the cell surface or circulating in human serum. Interestingly, previous studies reported that IF1 can switch between two different states: an active dimeric form and an inactive tetrameric form, depending on the pH [24], [25]. In our conditions, after SDS-PAGE and western blotting, we indeed detected recombinant IF1 both as a monomer and a dimer, suggesting that dimerization of IF1 was resistant to denaturing conditions. Identification of IF1 in human serum by mass spectrometry analysis was performed from digested fragments corresponding to the size of the monomeric IF1. Analysis of serum sample at the size of the dimer has not been conducted because serum proteins detected by colloidal blue staining were much more abundant at the size of the dimer, which would make IF1 peptides more difficult to detect. However, in immunoprecipitation experiments, we mainly detected IF1 in the serum at the size of the dimer, even if monomer can also be detected upon longer exposure times. This suggests that both monomeric and dimeric forms of IF1 are present in serum but IF1 seems to be mostly found as the active dimeric form, like in HepG_2_ cells. The formation of the IF1 dimer might be dependent on various parameters, such as pH conditions or post-translational modifications. The physiological pH of our samples for western blotting in all conditions is consistent with the presence of the dimers [25]. In addition, post-translational modifications could also favour or stabilize the formation of the dimers in serum. Furthermore, since the dimeric form of IF1 appears as a double band, we can hypothesize that IF1 is associated with other serum proteins. It is also conceivable that the double band reflects some post-translational modifications or alternative splicing of IF1. Indeed, alternative splicing occurs at IF1 gene locus, leading to distinct transcript variants. In human, three different isoforms have been identified. IF1 variant (NCBI Reference Sequence: NP_057395.1) encodes the longest isoform, while IF1 variant 2 (NCBI Reference Sequence: NP_835497.1) and variant 3 (NCBI Reference Sequence: NP_835498.1) have a shorter and distinct C-terminus compared to isoform 1. Mass spectrometry led to the identification of four peptides that only overlap with the C-terminus part of IF1 variant 1. However, one might speculate that different IF1 variants are present in human serum since our anti-IF1 antibody recognizes all these variants (data not shown). Therefore, further investigations are needed to better characterize the molecular components of dimeric IF1.

In this study, the first measurements of serum IF1 concentrations have been performed among normal healthy male adults, showing a normal distribution (95% confidence interval: 0.22–0.82 µg/mL). Moreover, HDL-cholesterol was significantly different according to IF1 distribution, a positive correlation being observed between both parameters (r = 0.259, p = 0.009). Interestingly, no relationship was found between IF1 levels and various environmental parameters known to affect HDL concentrations, such as cigarette smoking, alcohol consumption or physical activity. This suggests that IF1 could have an impact on HDL levels independently on those environmental factors. As reminded above, experiments in cultured hepatocytes and in perfused rat livers have demonstrated that exogenous IF1 impairs the hepatic uptake of HDL particles through the ecto-F_1_-ATPase/P2Y_13_ pathway [6]. In line with these observations, Giorgio *et al.* recently reported that, upon bile-duct ligation in rat, ecto-F_1_-ATPase activity was downregulated due to an increased expression level of endogenous IF1 at the cell surface [19]. Altogether these data suggest that IF1 might control hepatic HDL-cholesterol catabolism and it is tempting to speculate that IF1 would act similarly in humans leading to increased HDL-cholesterol levels. A comparable situation is encountered with hepatic lipase variants. Hepatic lipase, located in sinusoid capillaries, acts on HDL particles and stimulates their subsequent liver uptake [26]. Common genetic variants within the promoter result in a decreased *in vivo* hepatic lipase activity, repeatedly associated with elevated HDL levels [27]. Whether the 10% variation of HDL-cholesterol observed from both sides of IF1 median is relevant regarding cardiovascular risk protection needs further investigations. Only one other parameter, TG level and correlatively VLDL-cholesterol, was found different according to IF1 concentrations. So far, we have no evidence of a particular impact of IF1 on TG metabolism. However, components of the ATP synthase complex have been described at the surface of adipocytes and might participate to the control of lipid storage [28]. Also, a direct impact of IF1 on the metabolism of TG-rich lipoproteins cannot be excluded. Serum TG and HDL-cholesterol are inversely interrelated. Thus, further determinations of serum IF1 in larger populations and in various pathophysiological contexts are required, in order to assess whether or not IF1 can be considered as a new, independent determinant of HDL levels.

## Materials and Methods

### Study Population

The study population was approved by the local Ethics Committee of the hospital of Toulouse (CHU Toulouse/INSERM) and written informed consent was obtained from all participants involved in the study. Biological collection was constituted according to the principles expressed in the Declaration of Helsinki and registered under number DC-2008-403 at the Ministry of Research and at the Regional Health Agency.

Subjects were randomly selected from controls of a large case-control study carried out in the Toulouse area (France), by the Cardiovascular Epidemiology team of our University (Dr J.B. Ruidavets). They were all participants of a study on genetic, biological and environmental determinants of coronary artery disease (referenced as Genes study, [20]). In the frame of this study, 800 control male healthy subjects, aged 45–75, were randomly selected from the general population using electoral rolls. Subjects had an extensive evaluation of cardiovascular risk factors and clinical investigation included anthropometric variables, blood pressure measurements, measured twice at rest, and assessment of various metabolic parameters. Of this control population, 100 subjects were randomly drawn for serum IF1 measurements.

### Constructs, RNA interference and transfection

The sequence encoding the mature peptide of human IF1 (NCBI Reference Sequence: NM_016311.4), without the sequence of the mitochondrial targeting signal (MTS), was amplified by PCR from HeLa cell cDNA. The amplified fragment was then introduced at BamHI/HindIII restriction sites into VariFlex Expression vector pBEn-SBP-SET1-Qa (Stratagene) containing the Streptavidin Binding Peptide coding sequence (SBP tag). The resulting expression plasmid was verified by DNA sequencing and known as pBEn-SBP-IF1. In RNA interference (RNAi) experiments, cells were transfected twice at a 24 h interval using LipofectamineTM RNAiMAX transfection Reagent (Invitrogen) with 21-nucleotide RNA duplexes (siRNA Hs ATPIF1 6 from Qiagen), replated 4 h after the second transfection, and analyzed 36 h later.

### Recombinant protein

Recombinant human IF1 (rIF1) was produced in *E. coli* BL21 (DE3) strain. Briefly, the bacterial pellet was resuspended in lysis buffer (0.5 M NaCl, 0.7 M sucrose, 1 mM EDTA, 1% Triton X-100, 5 mM β-mercaptoethanol, Roche protease inhibitor cocktail) and then sonicated. The soluble proteins including rIF_1_ were diluted in binding buffer (50 mM ammonium carbonate, 0.5 M NaCl, pH 10.0), filtered through a 0.22 µm filter and then applied at 1 mL/min to a HiTrap™ Streptavidin HP 1 mL column (GE Healthcare). After washes, rIF1 was eluted in 6 M guanidine elution buffer. Pooled fractions containing rIF1 protein fused with SBP tag were dialyzed against thrombin-cleavage buffer (20 mM Tris-HCl pH 8.4, 150 mM NaCl, 2.5 mM CaCl_2_) and thrombin (10 U/mg protein) was added overnight at room temperature in order to cleave the SPB tag. The sample was then loaded onto HiTrap™ Streptavidin HP 1 mL column and pure IF1 was found in the flow-through fraction. rIF1 was >95% pure as determined by SDS-PAGE (data not shown).

### IF1 antiserum preparation

A polyclonal antibody was raised in New Zealand White rabbits against recombinant human IF1 (P.A.R.I.S, Compiègne, France). Total serum IgG were purified from serum by affinity chromatography using protein A Sepharose CL-4B columns (GE healthcare), according to manufacturer's instructions. Purity of IgG fractions was assessed by SDS-PAGE. Specific IgG against IF1 were purified from total serum IgG by affinity chromatography using IF_1_ column.

### SPR (Biacore) Analysis

Purified anti-IF1 antibody was immobilized by amine linkage on CM5 chips (Biacore AB) after NHS-EDC activation. Binding was analyzed in a Biacore 3000 apparatus. Increasing concentrations of rIF1 were injected at a flow rate of 20 µL/min, exposed to the surface for 175 s (association phase), followed by a 200 s flow running during which dissociation occurred. Between injections, in order to recover the prebinding baseline, the sensorchip surface was regenerated by a 5 µL injection of 0.01% SDS (15 s of contact time). The apparent *K*_d_ value was calculated with the CLAMP software [21].

### Immunofluorescence

HeLa cells cultured on coverslips were incubated or not with Mitotracker (Invitrogen) for 30 min at 37°C. Then, cells were fixed with 4% paraformaldehyde and permeabilized with 0.1% Triton X-100 for 60 min. After saturation of unspecific sites with PBS containing 10% foetal calf serum, cells were first incubated with the primary antibody (monoclonal anti-alpha ATP synthase from Invitrogen; polyclonal anti-IF1 antibody), then with fluorescently labeled secondary antibodies from Jackson Immunoresearch Laboratories (West Grove, PA). Pictures were captured using a Zeiss LSM 710 confocal microscope equipped with a 63× Plan-Apochromat objective.

### Immunoprecipitation

100 µL of serum from 10 individuals selected from the study population were pooled together. This serum pool was then pre-fractioned using the ProteoMiner™ kit (Bio-Rad Laboratories) according to the manufacturer's protocol. Immunoprecipitation of IF1 from human serum was performed using Pierce ® Crosslink Immunoprecipitation Kit (ThermoScientific) according to manufacturer's instructions. Briefly, 10 µg of pre-immune antibodies or anti-IF1 antibodies were covalently crosslinked onto protein A/G resin and then incubated overnight with 2 µg of recombinant IF1 (rIF1) or 500 µg of proteins either from serum or from HepG_2_ cell, after cell lysis in 150 mM NaCl, 1% Triton X-100, 20 mM Tris pH 7.6, protease inhibitor cocktail (Roche). After washes, the bound proteins were eluted in 0.2 M glycine pH 2.8 and 5× Laemmli sample buffer (200 mM Tris-HCl pH 6.8, 10% SDS, 50% glycerol, 125 mM DTT, 0.05% bromophenol blue) was added. Protein samples were then boiled for 5 min, loaded on 15% SDS-PAGE and transferred to nitrocellulose membrane, which was incubated at room temperature for 1 h in 5% non-fat dry milk in TBS (50 mM Tris, 150 mM NaCl) containing 0.05% Tween 20 (TBST). The membrane was incubated overnight at 4°C in TBST containing 1% non-fat dry milk and 1% BSA with our in-house developed anti-IF1 antibody at 1 µg/mL. The membrane was washed three times in TBST and incubated with anti-rabbit IgG-Horse Radish Peroxidase-conjugated antibody (1/4000) then rinsed in TBST and revealed for chemiluminescence (Sigma).

### Targeted analysis by Multiple Reaction Monitoring

500 µg of pre-fractioned serum proteins were diluted in Laemmli sample buffer as well as 5 µg of rIF1. Cysteine S-S bridges were reduced and alkylated by iodoacetamide and the samples were then loaded onto a 15% polyacrylamide gel. After Coomassie Blue staining, one major visible band corresponding to rIF1 monomer was cut. In parallel, a slice was cut at the same molecular weight size than rIF1 in serum sample lane. The gel slices were washed with 100 mM ammonium bicarbonate and then with 100 mM ammonium bicarbonate, acetonitrile (1∶1). Proteins were digested in gel by incubation with 0.6 µg of modified sequencing grade trypsin (Promega) in 50 mM ammonium bicarbonate at 37°C overnight. The resulting peptides were extracted by several incubations with acetonitrile and 10% formic acid, dried in a SpeedVac, and finally resuspended in 5% acetonitrile, 0.05% trifluoroacetic acid. The peptide mixture from rIF1 was used for optimisation of human IF1 detection by nanoLC/MS in Multiple Reaction Monitoring (MRM) mode using an Ultimate 3000 system (Dionex, Amsterdam, The Netherlands) coupled to a 5500 QTrap mass spectrometer (AB Sciex, Foster City, CA, USA). 1 pmol of rIF1 was loaded on a C_18_ precolumn (300-µm inner diameter×5 mm, Dionex, Amsterdam, The Netherlands) at 20 µL/min in 2% acetonitrile, 0.05% trifluoroacetic acid. After desalting, the precolumn was switched on-line with the analytical column (75 µm inner diameter×15 cm silica capillary tubing packed with Reprosil-Pur C18-AQ 3 µm phase, Dr. Maisch, GmbH, Germany) equilibrated in 100% solvent A (5% acetonitrile, 0.2% formic acid). Peptides were eluted using a 0–50% gradient of solvent B (80% acetonitrile, 0.2% formic acid) during 25 min at 300 nL/min flow rate. The 5500 QTrap was operated in MRM mode with the Analyst software (version 1.5.1, AB Sciex, Foster City, CA, USA). Four peptides of human IF1 giving the most intense signals were monitored in MRM with quadrupole Q1 and Q3 set to Unit resolution, optimal collision energies and declustering potentials were determined for three parent/fragment transitions of each peptide. During the run, full scan MS/MS acquisitions (EPI, Enhanced Product Ion) were also triggered when MRM signal exceeded 200 counts, with a mass tolerance of 250 mDa, the Linear Ion Trap (LIT) was set at 150 ms fixed fill time. A third of the peptide extract from serum sample was then loaded on the system in the same way than rIF1 and the analysis by the 5500 QTrap was performed in the same mode (MRM+EPI) with the optimized transition list. A blank run in the same conditions was performed before analyzing the serum sample in order to ensure the non-contamination of the nanoLC system by rIF1. The Mascot Daemon software (version 2.3.2, Matrix Science, London, UK) was used to perform database searches. To automatically extract peak lists from Analyst wiff files, the Mascot.dll macro (version 1.6b27) provided with Analyst was used and set for no grouping of MS/MS scans. Data were searched against *Homo sapiens* entries in the Uniprot protein database (September 2010 version – 96635 sequences). Carbamidomethylation of cysteines was set as a fixed modification and oxidation of methionines and deamidation of asparagines and glutamines were set as variable modifications for Mascot searches. Specificity of trypsin digestion was set for cleavage after lysine or arginine except before proline, and two missed trypsin cleavage sites were allowed. The mass tolerances in MS and MS/MS were set to 0.6 Da, and the instrument setting was specified as “ESI-QUAD”.

### Competitive ELISA

A competitive ELISA was devised in order to quantify IF1 in human sera. The wells of 96-well Polysorb ELISA plates (NUNC, Roskilde, Denmark) were coated with 50 ng of rIF1 in bicarbonate buffer (0.1 M, pH 9.6). Serum samples were thawed at room temperature and 50 µL of each patient serum, or rIF1 standard (0; 0.025; 0.050; 0.075; 0.1; 0.3; 1 and 2.5 µg/mL), were incubated with biotinylated anti-IF1 polyclonal antibody (dilution: 1/1000 in PBS; 0.05% Tween-20; 1% BSA) overnight at 4°C. The plates were washed and then incubated with blocking buffer (PBS buffer pH 7.4, 3% BSA) for 1 hour at room temperature. After washing, the sample/antibody mixtures were added and incubated for 4 hours at room temperature. The plates were washed again and incubated with streptavidin-HRP (dilution: 1/5000 in PBS pH 7.4; 0.05%Tween-20; 1% BSA) (Invitrogen, Cergy Pontoise, France) for 1 hour at room temperature. After washing, plates were incubated with horseradish peroxidase (HRP) substrate TMB (3,3′,5,5′-tetramethylbenzidine). The reaction was stopped with HCl 1N, and the plates were then read at 450 nm in a microplate reader (Varioscan Flash, Thermo electron corporation). The 570 nm optic density (background) was subtracted. Sample concentrations were determined using the standard curve (fit type: four parameter logistic).

### Serum lipid and glucose measurements

Blood was collected after an overnight fast. Serum total cholesterol, HDL-cholesterol (HDL-C), triglycerides (TG) and glucose were assayed with enzymatic reagents on an automated analyzer (Hitachi 912, Roche Diagnostics, Meylan, France). LDL-cholesterol (LDL-C) was calculated using the Friedewald formula, with VLDL-C (g/L) = TG (g/L)/5 as long as TG concentration is below 4 g/L [22].

### Statistical analysis

All results are presented as means and SD (Standard Deviation). Comparison between groups was made using two-tailed unpaired Student's t tests. A logarithmic transformation was done for triglycerides, because of skewed distribution. Pearson and Spearman coefficients were calculated to study correlations between IF1 serum level and HDL-C and TG serum levels respectively. Outcomes of p<0.05 were considered as statistically significant. Analyses were performed using GraphPad Prism 5 software (GraphPad Software, La Jolla, CA, U.S.A.).
